# A 5-year prospective study of health-related quality of life in irradiated head and neck cancer patients: three trends of HRQL over time

**DOI:** 10.1007/s00405-022-07789-7

**Published:** 2023-01-11

**Authors:** Susan Aghajanzadeh, Lisa Tuomi, Therese Karlsson

**Affiliations:** 1 Department of Otorhinolaryngology, Head and Neck Surgery, Institute of Clinical Sciences, Sahlgrenska Academy, University of Gothenburg, Gothenburg, Sweden; 2 Department of Otorhinolaryngology, Head and Neck Surgery, Region Västra Götaland, Sahlgrenska University Hospital, Gothenburg, Sweden

**Keywords:** Radiotherapy, Head and neck neoplasm, Quality of life

## Abstract

**Purpose:**

Head and neck cancer (HNC) is one of the fastest increasing cancer-types, where both disease and oncologic treatment have severe impact on health-related quality of life (HRQL). This study aimed to report HRQL prospectively up to 5-years following radiotherapy-treatment in HNC and to, if possible, identify trends in HRQL over time.

**Methods:**

This prospective study followed 211 patients receiving curatively intended radiotherapy pre-diagnosis, 3-, 6-, 12- and 60-months post-radiotherapy completion. HRQL was measured using EORTC QLQ-C30 and EORTC QLQ-HN35.

**Results:**

A deterioration three months post-radiotherapy was reported in 14/15 domains of EORTC QLQ-C30. Eight out of 12 domains had recovered to baseline-values at 12 months post-radiotherapy and remained unchanged up to study endpoint. Corresponding figures for EORTC QLQ-HN35 were deteriorations in 15/16 domains at three months post-radiotherapy, with recovery of 5 domain at 12-months, whereas the other 11 domains remained significantly worse at 5-years post-RT compared to baseline.

**Conclusion:**

Following the deterioration in HRQL seen immediately following radiotherapy, the continued course of HRQL can be divided into three trends: short-term deterioration, long-term deterioration and long-term improvements. The combination of disease- and diagnosis-specific questionnaires is crucial when assessing HRQL in the HNC population.

## Introduction

In 2020, malignancy of the head and neck (HNC) accounted for 8% of all cancer worldwide [1]. In Sweden, it is one of the fastest increasing types of cancer [2], with survival rates improving due to early diagnosis as well as technical advances allowing more effective targeted treatments. Thus, survival as a measure of effective cancer treatment has over the past two decades been complemented with the concept of health-related quality of life (HRQL) [3].

HNC may be treated by radiotherapy (RT), chemotherapy and surgery or a combination thereof, resulting in both short- and long-term complications including trismus, dysphonia, dysphagia, pain and xerostomia [4–6]. The course of HRQL following oncologic treatment has been intensely studied short-term, revealing a marked deterioration in HRQL following treatment with some recovery within the first year [7, 8]. To date, only a handful of studies have investigated effects of HRQL long-term, i.e., up to 5 years showing that some symptoms worsen over time whereas other functions improve [9–11]. These studies are sometimes hampered by a small population at study endpoint or include both surgical and radiotherapeutic treatment modalities, resulting in heterogenicity which may lead to interpretation difficulties.

Hence, this study aimed to report HRQL prospectively up to 5 years following treatment by curatively intended radiotherapy in HNC and to, if possible, identify trends in HRQL over time.

## Material and methods

### Study participants

Patients diagnosed with primary HNC in the western part of Sweden, who were to receive curatively intended radiotherapy, were asked to participate in the study. Inclusion criteria encompassed being at least 18 years of age, receiving curatively-intended radiotherapy with or without chemotherapy and a tumour site in the oral cavity, oropharynx, paranasal sinuses and nasal cavity, nasopharynx or cervical carcinoma of unknown primary (CCUP). Exclusion criteria was recurrent disease, a primary surgical treatment, a performance status or mental capacity too poor to partake in examinations. As the study was part of a study examining trismus, patients were also excluding if they had a tumour site where trismus was not expected to develop, i.e., laryngeal or hypopharyngeal. Comorbidity was measured using the Adult Comorbidity Index evaluation (ACE-27) [12, 13].

### Study design

Patients were followed prior to radiotherapy (baseline), 3-, 6-, 12-, and 60-months post-completion of radiotherapy. At each time-point, they completed validated questionnaires designed to measure HRQL.

### Oncologic treatment

Traditional curative radiotherapy was given according to regional guidelines. Accelerated hyperfractionated radiotherapy was administered to 109 patients (52%), consisting of 1.7 Gray (Gy) doses given twice daily, five days/week to a total of 64.6 Gy. The remaining patients (*n* = 102, 48%) received accelerated fractioning 1–2 times daily in 2–2.4 Gy fractions, five days/week totaling 64–68 Gy. Sixty-seven percent (*n* = 141) received additional chemotherapy according to regional guidelines with 5-fluorouracil or cisplatin.

### European organization for research and treatment of cancer

The European Organization for Research and Treatment of Cancer (EORTC) Quality-of-Life Questionnaire Core-30 (QLQ-C30) is a questionnaire developed to evaluate HRQL for the general cancer population. The QLQ-C30 consists of 30 items focusing on physical and psychosocial functioning as well as symptoms that cancer patients experience [14]. A disease-specific head and neck (HN) module has been developed to complement this (EORTC QLQ-HN35) and consists of 35 items addressing symptoms specific to head and neck cancer and its treatments [15]. Scores range from zero to 100. Higher scores for the functioning domains and Global quality of life represent better function, whereas higher scores for symptom domains and single items indicate a higher symptom burden.

### Statistical analysis

Number and percentages are presented for categorical variables, whilst continuous variables are presented using mean and standard deviation (SD). For comparisons between groups, Fischer’s Exact test was used for dichotomous variables, Mantel–Haenszel Chi Square test for ordered categorical variables, Chi Square test for non-ordered categorical variables and Mann–Whitney *U* test for continuous variables. For changes over time within groups Wilcoxon signed rank test was used for continuous variables. All tests were two-sided and conducted at a 5% significance level. The SAS version 9.4 for PC was used for analyses.

### Ethical considerations

The study was conducted according to the Declaration of Helsinki and was approved by the Regional Ethic Review Board in Gothenburg, Sweden. All participants gave their written informed consent before study inclusion.

## Results

### Sociodemographic data

A total of 211 patients were included in the study between the years 2007–2012. The clinical and sociodemographic characteristics of the cohort at baseline are outlined in Table 1. At the study endpoint, 60 months post-RT, 129 (61%) were still eligible for evaluation, whereas 82 had dropped out. Reasons for ended study participation were deceased (*n* = 35), reduced general health (*n* = 8) and unknown (*n* = 39).

**Table 1:** Sociodemographic data at baseline (mean, *n*, SD, %) for the entire cohort compared to those completing the study

	All patients (*n* = 211)	Patients completing the study (*n* = 129)	*p* value
Age	61.0 (10.2)	59.5 (9.7)	
Gender			
Male	157 (74.4%)	92 (71.3%)	
Female	54 (25.6%)	37 (28.7%)	0.26
Living alone			
Yes	153 (72.5%)	30 (23.2%)	
No	58 (27.5%)	99 (76.7%)	0.099
Number of school years			
6–9 years	58 (27.5%)	30 (23.3%)	
9–12 years	99 (46.9%)	60 (46.5%)	
> 12 years	54 (25.6%)	39 (30.2%)	0.027
Employment			
Full time	98 (46.4%)	68 (52.7%)	
Part time	16 (7.6%)	11 (8.5%)	
Unemployed	8 (3.8%)	3 (2.3%)	
Old age pensioner	70 (33.2%)	33 (25.6%)	
Early retiree	19 (9.0%)	14 (10.9%)	0.017
Smoking			
Yes	49 (23.2%)	100 (77.5%)	
No	162 (76.8%)	29 (22.5%)	1.00
Karnofsky Performance Scale*	97.5 (6.6)	98.7 (5.1)	< 0.001
BMI (mean)			
< 18.5	26.6 (4.7)	26.7 (3.8)	
18.5–25	3 (1.4%)	2 (1.6%)	
> 25	68 (32.4%)	37 (28.7%)	
Missing = 1	139 (66.2%)	90 (69.8%)	0.28
Tumour stage			
I	8 (3.8%)	6 (4.7%)	
II	36 (17.1%)	23 (17.8%)	
III	49 (23.2%)	30 (23.3%)	
IV	118 (55.9%)	70 (54.3%)	0.53
Tumour site			
Oral cavity	34 (16.1%)	15 (11.6%)	
Oropharynx	132 (62.5%)	84 (65.1%)	
Salivary gland	8 (3.8%)	5 (3.9%)	
Nasopharynx	16 (7.6%)	10 (7.8%)	
CCUP	21 (10.0%)	15 (11.6%)	0.24
Cancer treatment			
Only RT	70 (33.1%)	41 (31.8%)	
RT + Chemo	141 (66.9%)	88 (68.2%)	0.45
ACE27			
No comorbidity	92 (43.6%)	64 (49.6%)	
Mild comorbidity	76 (36.0%)	44 (34.1%)	
Moderate comorbidity	35 (16.6%)	19 (14.7%)	
Severe comorbidity	8 (3.8%)	2 (1.6%)	0.006

*RT* radiotherapy, *CCUP* cervical carcinoma of unknown primary, *Chemo* chemotherapy, *SD* standard deviation, *ACE27* Adult Comorbidity Evaluation 27

*Range from 0 to 100 equals perfect health

### EORTC QLQ-C30

Data from the EORTC QLQ-C30 is presented in Table 2. A deterioration three months post-RT was reported in all items with the exception of *Emotional functioning*, which continued to improve throughout the study period. At 60 months post-RT, only *Physical functioning* and *Dyspnoea* were significantly worse compared to baseline. Eight out of 12 domains had recovered to baseline-values at 12 months post-RT and remained unchanged up to study endpoint. Between 12- and 60 months only three domains showed statistically significant changes (Physical functioning, Dyspnoea, Insomnia).

**Table 2:** EORTC QLQ-C30

	Pre-RT, *N* = 211	3 months post-RT, *N* = 199	6 months post-RT, *N* = 189	12 months post-RT, *N* = 180	Δ pre-RT—12 months *p* value	60 months post-RT N = 129	Δ pre-RT—60 months *p* value
	Mean (SD)	Mean (SD)	Mean (SD)	Mean (SD)		Mean (SD)	
Functioning domains							
Physical function	89.7 (16.8)	78.4 (8.9)	82.8 (19.0)	86.6 (16.9)	< 0.001	83.9 (21.6)	< 0.001
Role function	74.2 (33.9)	61.0 (34.5)	72.2 (29.7)	77.8 (29.0)	0.21	81.7 (28.4)	0.27
Emotional function	70.1 (24.0)	76.9 (22.7)	79.9 (22.0)	81.5 (22.6)	< 0.001	84.1 (20.8)	< 0.001
Cognitive function	85.0 (18.3)	81.8 (21.5)	85.1 (18.9)	85.0 (20.4)	0.94	83.7 (19.8)	0.05
Social function	82.9 (23.6)	68.7 (30.6)	76.8 (26.3)	80.5 (27.0)	0.26	83.5 (24.8)	0.82
Global QL	65.8 (20.9)	61.1 (20.6)	66.5 (20.3)	68.9 (20.9)	0.10	71.6 (24.8)	0.07
Symptom Scales							
Fatigue	23.0 (22.2)	39.2 (26.5)	30.8 (25.2)	26.2 (24.4)	0.29	24.9 (29.1)	0.16
Nausea/vomiting	4.57 (12.4)	9.2 (16.3)	4.3 (11.7)	4.5 (12.5)	0.66	4.2 (13.2)	0.85
Pain	23.7 (26.4)	27.1 (25.8)	21.0 (24.4)	17.7 (23.1)	< 0.01	17.5 (26.8)	0.08
Single items							
Dyspnoea	15.5 (24.3)	29.2 (29.6)	23.0 (26.2)	17.5 (22.6)	0.48	20.2 (24.7)	< 0.01
Insomnia	27.9 (29.3)	28.2 (30.0)	21.6 (27.7)	19.3 (28.7)	< 0.001	22.7 (29.2)	0.37
Appetite loss	12.9 (23.6)	37.4 (35.1)	23.9 (32.0)	19.5 (29.8)	< 0.01	9.5 (20.6)	0.27
Constipation	8.5 (19.6)	17.4 (26.5)	11.1 (22.3)	9.9 (20.3)	0.54	10.3 (21.3)	0.15
Diarrhoea	4.3 (12.6)	7.6 (16.4)	6.0 (15.1)	5.2 (15.8)	0.74	5.7 (17.4)	0.25
Financial loss	13.5 (25.0)	23.9 (31.5)	17.8 (30.3)	17.0 (28.4)	< 0.05	9.9 (23.6)	0.53

*RT* radiotherapy, *SD* standard deviation, *QL* quality of life

### EORTC QLQ-HN35

Data from EORTC QLQ-HN35 is presented in Table 3. A deterioration three months post-RT was reported in 15 out of 16 domains. Five of these domains (Pain, Social contact, Sexuality, Cough and Felt ill recovered to pre-RT values at 12 months, whereas the other 11 domains remained significantly worse at 5-years post-RT compared to baseline. Only domains Dry mouth and Cough showed a significant change between 12 months and 5 years post-RT.

**Table 3:** EORTC QLQ-HN35

	Pre-RT, *N* = 211	3 months post-RT, *N* = 199	6 months post-RT, *N* = 189	12 months post-RT, *N* = 180	Δ pre-RT—12 months *p* value	60 months post-RT, *N* = 129	Δ pre-RT—60 months *p* value
	Mean (SD)	Mean (SD)	Mean (SD)	Mean (SD)		Mean (SD)	
Symptom domains							
Pain	21.9 (22.4)	33.9 (25.7)	27.7 (22.1)	22.7 (21.5)	0.43	17.3 (21.8)	0.18
Swallowing	14.4 (21.6)	29.7 (27.1)	22.9 (23.4)	21.1 (22.7)	< 0.001	18.6 (24.3)	< 0.01
Senses	9.1 (18.4)	37.4 (29.5)	33.2 (28.1)	29.6 (27.2)	< 0.001	21.8 (26.2)	< 0.001
Speech	12.3 (18.7)	21.2 (19.3)	17.9 (19.4)	15.3 (19.0)	< 0.05	13.9 (20.6)	0.02
Social eating	13.2 (20.8)	36.2 (29.0)	29.2 (25.8)	26.6 (29.6)	< 0.001	17.8 (23.9)	< 0.01
Social contact	7.1 (15.8)	11.9 (18.6)	9.0 (15.7)	8.8 (17.4)	0.18	9.1 (18.0)	0.10
Sexuality	28.3 (35.9)	46.5 (37.1)	37.3 (36.1)	31.4 (33.9)	0.24	33.0 (34.6)	0.06
Symptoms single items							
Teeth	7.5 (19.1)	17.1 (28.7)	22.0 (31.0)	19.7 (29.1)	< 0.001	22.4 (33.8)	< 0.001
Open mouth	8.7 (20.7)	24.0 (27.7)	27.3 (28.1)	22.9 (27.6)	< 0.001	22.2 (31.0)	< 0.001
Dry mouth	18.1 (27.2)	74.7 (30.0)	73.3 (28.6)	65.7 (31.5)	< 0.001	55.7 (33.3)	< 0.001
Sticky saliva	16.5 (26.0)	57.4 (36,4)	53.7 (36.2)	54.0 (35.6)	< 0.001	45.3 (43.7)	< 0.001
Cough	16.9 (23.4)	25.4 (28.2)	25.7 (29.4)	20.9 (26.9)	0.10	22.8 (28.8)	< 0.01
Felt ill	18.3 (27.2)	23.7 (28.1)	17.0 (25.0)	15.6 (25.3)	0.45	11.9 (22.1)	0.13
Pain killers	53.4 (50.0)	51.0 (50.1)	36.5 (49.4)	33.5 (47.3)	< 0.001	30.2 (47.8)	< 0.001
Nutritional supp	12.5 (33.2)	44.3 (49.8)	39.2 (49.0)	22.7 (42.0)	0.001	16.7 (37.4)	< 0.05
Feeding tube	2.4 (15.4)	30.6 (46.2)	13.7 (34.5)	8.0 (27.1)	< 0.05	8.8 (28.4)	< 0.05

*RT* radiotherapy, *SD* standard deviation

### Patients not completing the 60-month follow-up

Patients who were eligible for evaluation at the study endpoint were, at baseline, significantly younger (59 vs 64, *p* = 0.009), had higher, i.e., better, Karnofsky performance scores (98 vs 96 *p* ≤ 0.001) and suffered from less comorbidity according to the Adult Comorbidity Evaluation 27 (ACE-27, *p* = 0.006) compared to those who dropped out. They also reported better scores on EORTC QLQ C30 Physical functioning (92 vs 85, *p* = 0.002), Role functioning (79 vs 67, *p* = 0.016), EORTC QLQ HN35 Pain (19 vs 27, *p* = 0.018), Swallowing (12 vs 19, *p* = 0.026), Senses (6 vs 14, *p* = 0.003), Speech (9 vs 17, *p* = 0.003) and Social eating (11 vs 17, *p* = 0.044).

## Discussion

This prospective study reports HRQL for HNC patients receiving curative radiotherapy up to five years post-RT completion, with one of the largest remaining cohorts at study endpoint.

In general, HRQL deteriorated following RT with worse HRQL outcomes reported across nearly all domains and single items at three to six months post-RT—a trend which has also been reported by Hammerlid et al. [16] and Nyqvist et al. [10]. However, following this initial reduction, three trends of HRQL were identified, i.e., short-term deteriorations, long-term deteriorations and domains that continuously improved.

Firstly, short-term deteriorations in HRQL were seen when recovery to pre-RT levels at 12 months post-RT occurred and was observed in a majority of the EORTC QLQ-C30 domains (75%, 8/12). Less of the EORTC QLQ HN35 domains improved to pre-RT levels at 12 months (31%, 5/15). One explanation for these observed trends may be the presence of short-term adverse effects of oncologic treatment, which is better captured by the EORTC QLQ HN35. This hypothesis is widely supported by a majority of prospective HRQL studies in HNC [8–10, 16].

Long-term deteriorations, i.e., where the initial HRQL-reduction remained over time were found mainly in the diagnosis-specific HN35-module. Here, a continued deterioration compared to pre-RT was found at 5 years post-RT in the majority of items (11/16), probably reflecting the permanent tissue injury caused by radiotherapy [17]. In contrast, on the EORTC QLQ C30 only 2/12 items (Physical functioning and Dyspnoea) were significantly worse at study endpoint. This decline may well be explained by the increasing age of the study population. However, it also highlights the different nature of the two HRQL instruments, with the HN35-module being specific to symptoms related to HNC and the potential toxic effects of radiotherapy [17], whereas the C30-module is a general measure of well-being within a cancer population. Patients may learn to adapt to their overall novel situation, a concept known as response shift [18]. This is reflected by more reported improvements at 12 months post-RT in the C30-module, which is a more general and broad cancer HRQL instrument, despite experiencing a high symptom-specific burden as revealed by the HN35-module.

The final but less common trend was that of continuous improvement, which was only seen in the C30 domain Emotional functioning. There are conflicting reports regarding this in existing literature, where Hammerlid et al. found an identical trend in their 133 HNC patients three years post-RT [16]. Verdonck de Leeuw et al. only found improvements at 6 weeks post-RT, which then deteriorated up to 2-year post-treatment, albeit results from this study are difficult to interpret [8]. An improvement in mental health is, nonetheless, not surprising despite the physical deterioration and may be explained by tumour burden removal and patient survival.

Additionally, this study reveals that there was little improvement, or dynamic in general, between 1- and 5-years post-RT. When compared to Swedish population-based reference values [19], reported HRQL values were not strikingly different at the 5-year follow-up when examining EORTC QLQ C30-scores. However, when data from EORTC QLQ HN35 was compared, HNC patients reported worse scores in every domain compared to the norm population, again highlighting the importance of the combined use of questionnaires.

The strengths in this study lie in its prospective long-term follow-up with a large number of remaining patients at study endpoint. Nevertheless, it is important to be aware of selection bias as patients enrolled at the 5-year follow-up were younger and healthier pre-RT. It is also limited by the exclusion of patients with laryngeal and hypopharyngeal tumour sites, as patients with laryngeal cancer often report better HRQL-scores compared to other HNC-sites whereas patients with pharyngeal tumours generally report worse scores [7]. Finally, it should be highlighted that the fractionation protocol used in the study is not standard treatment protocol today but was at the time of inclusion and may thus decrease the generalizability of results somewhat.

### Clinical implications

The clarification of these trends allows clinicians to better inform patients of the course of symptoms as well as associated HRQL following treatment. It also highlights the importance of using combined questionnaires when measuring HRQL as valuable information may otherwise be omitted and can be used to tailor treatment according to symptomatology. As it is evident that the HNC population report radiotherapy-related complications for as long as 5 years post-RT, the need for rehabilitative programmes also becomes exceedingly important in post-cancer care in order to maintain as well as improve long-term HRQL for cancer survivors.

## Conclusions

Following the deterioration in HRQL seen immediately following radiotherapy, the continued course of HRQL can be divided into three trends: short-term deterioration, long-term deterioration and long-term improvements. Little dynamic in scores is seen between 1- and 5-years following treatment. The combination of disease- and diagnosis-specific questionnaires is crucial when assessing HRQL in the HNC population.
